# LPAI—A Complete AIoT Framework Based on LPWAN Applicable to Acoustic Scene Classification Scenarios

**DOI:** 10.3390/s22239404

**Published:** 2022-12-02

**Authors:** Xinru Jing, Xin Tian, Chong Du

**Affiliations:** 1 Shanghai Advanced Research Institute, Chinese Academy of Sciences, Shanghai 201210, China; 2 University of Chinese Academy of Sciences, Beijing 100049, China

**Keywords:** AIoT, domain adaptation, edge intelligence, LPWAN

## Abstract

Deploying artificial intelligence on edge nodes of Low-Power Wide Area Networks can significantly reduce network transmission volumes, event response latency, and overall network power consumption. However, the edge nodes in LPWAN bear limited computing power and storage space, and researchers have found it challenging to improve the recognition capability of the nodes using sensor data from the environment. In particular, the domain-shift problem in LPWAN is challenging to overcome. In this paper, a complete AIoT system framework referred to as LPAI is presented. It is the first generic framework for implementing AIoT technology based on LPWAN applicable to acoustic scene classification scenarios. LPAI overcomes the domain-shift problem, which enables resource-constrained edge nodes to continuously improve their performance using real data to become more adaptive to the environment. For efficient use of limited resources, the edge nodes independently select representative data and transmit it back to the cloud. Moreover, the model is iteratively retrained on the cloud using the few-shot uploaded data. Finally, the feasibility of LPAI is analyzed, and simulation experiments on the public ASC dataset provide validation that our proposed framework can improve the recognition accuracy by as little as 5% using 85 actual sensor data points.

## 1. Introduction

Low-Power Wide Area Network (LPWAN) is a wireless network that is an essential part of wireless communication for the Internet of Things (IoT) [1] and has received extensive attention in recent years [2]. LPWAN is designed to allow low-power and long-range transmission, which makes LPWAN have the following features. First, it has strict transmission rate and payload length limitations. Second, the edge nodes in LPWAN are required to operate for long periods without battery replacement, and are usually ultra-low-power embedded devices with about a few megabytes of memory resources, thus they have extremely limited computing resources compared to mainstream lightweight networks with millions of parameters [3]. Moreover, low-power microcontrollers use MHz-level frequencies, while mobile devices employ GHz-level frequencies, so their computational resources are also scarce. Among all LPWAN technologies, LoRa is the most widely used [4]. Unlike other solutions, LoRa is deployed in unlicensed bands so that users can build networks independently. As a result, anyone can have complete ownership and control of the LoRa network at a low operational cost.

LPWAN has been used in medical treatment [5,6] and traffic monitoring [7], especially in acoustic scene classification (ASC) scenarios. ASC refers to recognizing different indoor and outdoor scenarios via acoustic signals recorded by sensors. Many studies utilize AIoT technologies to optimize the performance of ASC tasks [8]. Zualkernan et al. [9] have designed a system for monitoring bat species based on echolocation audio. For scenarios such as wildlife monitoring, which requires the long-term deployment and challenging maintenance, LPWAN is the most effective network option. Implementing AIoT technology in these acoustic scenarios allows offloading computational tasks to nodes located at the edge of the network, thus effectively reducing the amount of data transmission in the medium and extending the lifetime of the edge nodes, which aligns with the needs of LPWAN. Subsequently, this brings several benefits [10,11], including ultra-low latency [12], reduced power consumption [13,14] and improved network reliability and data security [14,15].

However, due to the transmission characteristics of LPWAN, there remain challenges to the implementation of AIoT technologies in LPWAN. In particular, it is difficult for the cloud to utilize data from the sensors for model updates after deploying intelligent models, resulting in still poor recognition performance of edge nodes. This occurs due to several reasons.

- The domain shift problem exists between the environment and the data available in the cloud. Domain shift is caused by sensor type changes and deployment locations, resulting in differences between source and target domains, thus weakening the intelligence capabilities of the edge nodes.
- It is costly for LPWAN to upload a single packet of raw data back to the server. For example, the LoRa payload comprises only about a hundred levels of bytes. Capturing a 1-second voice recording at 16Khz can produce 64K bytes of raw data. Therefore, hundreds of LoRa packets would need to be sent to deliver them all to the cloud, which creates a huge overhead.
- From a data volume perspective, existing mobile-network-based frameworks support the backhaul of all target domain data, which is almost impossible for LPWAN. Moreover, even if only a small amount of data is uploaded, it is ineffective to retrain based on these few-shot samples using traditional methods.

There has been much research on AIoT frameworks in recent years. Huawei Technologies Co. et al. [16,17,18] proposed mobile device frameworks designed for high-dimensional data such as images, which are not suitable for the more restrictive LPWAN. Other frameworks, including Chiu et al. [19], Zualkernan et al. [9] and Chang et al. [20] are based on LPWAN; however, these studies do not consider updating the intelligence model according to the environment. They have still not implemented edge intelligence for LPWAN in this sense.

To increase the suitaility of LPWAN edge nodes for the environment, we propose LPAI, the first generic framework for implementing AIoT technology based on LPWAN applicable to ASC scenarios. LPAI not only realizes the intelligence of edge nodes in LPWAN, but more importantly, it allows resource-constrained edge nodes to continuously improve their performance using raw data, thus becoming more adaptive to the environment. The main contributions can be summarized as follows:

- LPAI enables the intelligence of edge nodes and reduces bias between the source and the target data. This enables edge nodes to be applied in real-world scenarios and continuously utilize unlabeled target data from the environment to improve their recognition performance.
- A data screening mechanism suitable for LPWAN is designed to improve the recognition performance of edge nodes. In this mechanism, edge nodes make independent decisions and only upload compressed features that help improve performance. Furthermore, iterative retraining is performed in the cloud based on few-shot compressed features returned by the nodes.
- We evaluate multiple datasets in ASC scenarios, proving that LPAI can become a general AIoT framework of LPWAN applicable to the ASC scenarios.

## 2. Related Work

### 2.1. Domain Adaptation

In computer vision, domain shift problems are solved using domain adaptation (DA) techniques. With DA techniques, the bias caused by the target data collected by actual sensors can be better mitigated, forcing the data distributions of the two domains to be similar [21,22,23,24,25,26,27,28,29]. Researchers have gradually veered toward wearables [30] and mobile devices [31,32], but rarely toward the more restrictive LPWAN.

### 2.2. AIoT Frameworks

We summarize the research on AIoT frameworks for mobile devices and LPWAN, as shown in Table 1. Huawei Technologies Co. [16] proposed an open-source deep learning training and inference framework called MindSpore. E. Raj et al. [17] implemented a deep learning model for human pose estimation and tracking based on MindSpore in computer vision. Rong et al. [18] designed a collaborative computing platform between edge devices and the cloud to support continuous model evolution and system updates. The foregoing studies are based on mobile networks. They support the uploading of all target domain data regardless of resources. Chiu et al. [19] propose an AIoT precision feeding management system based on LoRa network to improve the existing automatic feeding system in the mark. Chang et al. [20] proposed an intelligent assistive system based on wearable smart glasses and smart cane. However, LPWAN is only responsible for transmitting GPS information of where a fallen person is located. The wearable smart glasses implement the intelligence task.

## 3. Methods

### 3.1. Overview

**Figure 1:**
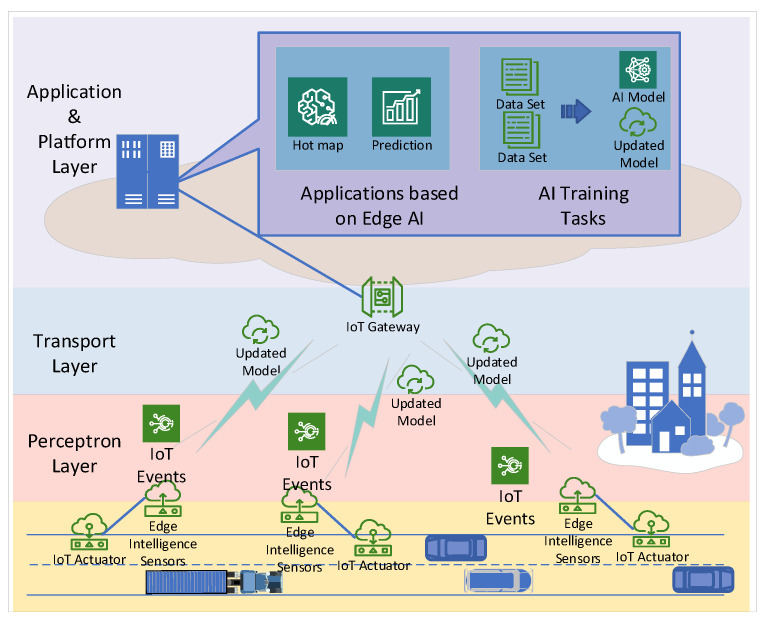
The overall framework of LPAI. It is divided into four layers, namely the application layer, platform layer, transport layer and perception layer.

LPAI is a general AIoT framework based on LPWAN for acoustic scene classification scenarios. Like the traditional AIoT framework, LPAI contains four layers from top to bottom: the application, platform, transport, and perception. The overall framework diagram of LPAI is in Figure 1. The application layer contains urban acoustic scenarios. Acoustic sensors are deployed at the entrance of construction sites to monitor incoming and outgoing traffic flow during unconventional hours, effectively discouraging illegal site operations. In this scenario, the input is the wave data collected by the edge nodes through acoustic sensors. After model inference, the output is the scene which the acoustic data belong to, such as traffic or pedestrian. Subsequently, the edge node determines whether the current data need to be uploaded by our proposed data screening algorithm. When a certain amount of data from the target domain is collected in the cloud, it is retrained with our cloud-based retraining algorithm. A new model that is more applicable to the environment is eventually obtained, thus continuously improving the recognition performance of nodes in real-world applications.

In LPAI, the platform layer comprises the cloud platform and operating system that handles complex intelligent tasks. The transport layer is mainly responsible for uplink and downlink data transmission. Furthermore, the perceptron layer is responsible not only for sensing, execution, and control, but also for executing small AI computational tasks and making independent decisions.

### 3.2. The Transport Layer

The transport layer is first introduced to describe the data flow of the framework. The transport layer is based on the standard LPWAN protocol to ensure generality of the LPAI. The transmission task is divided into uplink and downlink, as summarized below.

- The uplink uploads from the edge side to the cloud. To save resources, it mainly uploads compressed features of raw data from the environment and the statistical events of edge nodes.
- The downlink includes the model to be updated and the spatial distribution of the source domain, providing the basis for nodes to filter the data independently.

**Figure 2:**
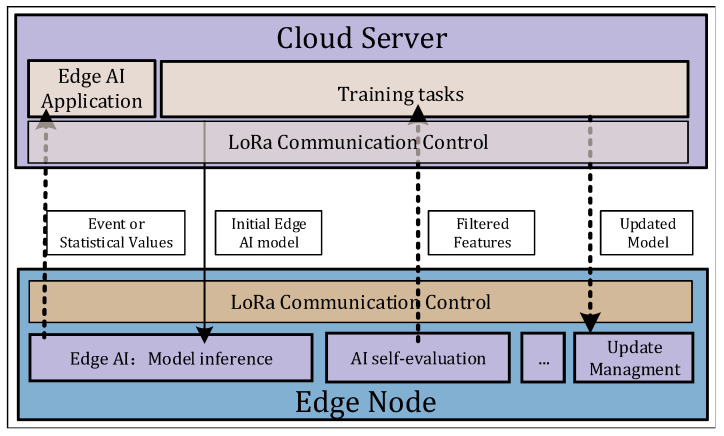
Data flow in the transport layer. In LPAI, the transport layer is mainly responsible for uplink and downlink data transmission. The arrows in the figure represent the data flow. The solid black line represents the initialization model that needs to be deployed locally on the node before the system operates, and the dashed line represents the data transmitted through the LPWAN protocol after deployment. Among them, the compressed features processed by the edge node and the response events are uploaded in the uplink, and the model to be updated is transmitted in the downlink.

In LPAI, the complete data flow process after implementing edge intelligence is as follows. An edge node periodically uploads the statistical information to the cloud to notify when a vehicle is detected. At the same time, it only uploads the compressed features that are more helpful for model updates. After a certain number of compressed features are uploaded, a new model is generated in the cloud. The newly produced model will then be sent down to the edge node via the downlink of the LPWAN protocol. Thus, a closed-loop complete framework is constructed. The complete data flow process is shown in Figure 2.

### 3.3. The Platform Layer

**Figure 3:**
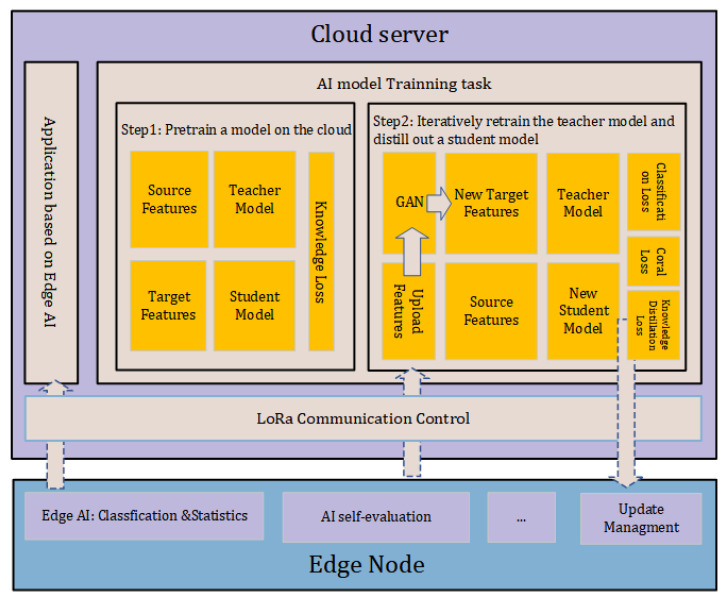
Tasks on the cloud. The task is divided into two steps, the first step is to train an initial model $S_{init}$ to implement edge intelligence. The second step is to retrain a teacher model with the target domain features, and then distilling a student model. Finally, the student model is redistributed to the nodes.

In LPAI, the platform layer provides complex computing tasks in the cloud. Its tasks are listed below, and are also shown in Figure 3:

- Generate a model that can classify acoustic scenes to enable edge intelligence for LPWAN.
- Perform a cloud-based retraining algorithm that can receive features from the target domain and retrain a new model using both the source domain and unlabeled target domain features.
- Describe the spatial distribution of the existing source features.

#### 3.3.1. Training an Initial Model

Initially, a portion of the labeled source features is on the cloud. This part of the source features needs supervised training to get an initial model $M_{init}$. Unlike traditional deep learning, this model performs simple classification tasks, usually two- [35], three- or four-class problems. In general, the more complex the classification task, the higher the training cost. Subsequently, $M_{init}$ can then be compressed and prepared for deployment on resource-constrained devices.

According to Hinton’s knowledge distillation method [36], a deep and complex network can transfer to a small and shallow network, also known as the teacher–student model. Inspired by the recent model distillation technique, we design a soft target loss in order to transfer novel knowledge. Considering an example $x_{i}^{t}\in D$, the teacher network can produce class probabilities by a softmax layer that converts its logits $z_{i}=\left\{z_{i}^{1},\ldots ,z_{i}^{K}\right\}$ to an output of probability $y_{i}$. The temperature *T* is put into the standard softmax function as shown in Equation (1), where *T* is a parameter denoting temperature, set to 1 in a standard softmax. It converts the logit values to pseudo-probabilites. The teacher model’s knowledge is transferred to the student model through Equation (4), using cross-entropy as the loss function, where $L_{soft}$ in Equation (2) refers to the cross entropy between the soft labels of the teacher $p_{j}^{T}$ and the student $q_{j}^{T}$ when the temperature is *T*. The higher the temperature, the greater the attention paid to negative labels. Since the teacher’s judgment is not entirely correct, $L_{hard}$ in Equation (3) is introduced to prevent overfitting. It represents cross-entropy between the student’s soft and hard labels at temperature T=1. When the teacher’s judgment is incorrect, the student learns by referring to the correct label. Finally, the student model $S_{init}$ is distributed to the nodes.

(1) $$y_{i}\left(x|t\right)=\frac{e^{\frac{z_{i}\left(x\right)}{T}}}{\sum_{j}e^{\frac{z_{j}\left(x\right)}{T}}}$$

(2) $$L_{soft}=-\sum_{j}^{N}p_{j}^{T}\log\left(q_{j}^{T}\right)$$

(3) $$L_{hard}=-\sum_{j}^{N}C_{j}\log\left(q_{j}^{{}^{\prime }}\right),\;C_{j}\in \left\{0,1\right\}$$

(4) $$L_{KD}=\alpha L_{soft}+\left(1-\alpha \right)L_{hard}$$

#### 3.3.2. Cloud-Based Retraining Algorithm

**Figure 4:**
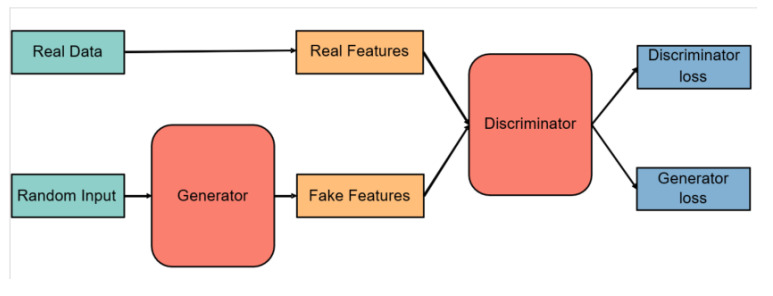
The structure of GAN. The generator is responsible for generating corresponding instances to deceive the discriminator. In this way, samples can be generated from a given domain. Real features represent the newly uploaded target domain features.

Because the number of newly uploaded target features is much smaller than the existing source domain, a feature-specific augmentation method is required to prevent poor generalization performance. The classical methods used for the augmentation of acoustic are usually specific to the raw data [37,38], not the features. Generative Adversarial Networks (GAN) are a popular research area in computer vision. GAN can expand target features by creating new features using its generator. Moreover, they can also achieve excellent performance in the case of few-shot learning. The structure of GAN is displayed in Figure 4. In the retraining phase, the training and test sets are the source domain $f_{s_{i}}$ and the expanded target features $f_{new\_t_{i}}$.

A pseudo-label is added to each unlabeled target feature in each training epoch. Because these pseudo-labels are still unreliable, features with confidence greater than $\gamma_{server}$ are selected iteratively and fed into the neural network. As the epoch increases, the model performance will improve, and the number of target features involved in training will increase, further enhancing the overall recognition performance. In a previous work, many loss functions were designed to decrease the distance between the source and target domains, such as MMD [39]. In LPAI, a simple coral loss [40] is used based on the two-norm distance of the covariance matrix between the two domains. As in Equation (5), *d* represents the batch size of each round of training. The source and target domain covariance matrices calculate as Equations (6) and (7), where 1 represents a column vector. This part is shown in Algorithm 1.

(5) $$L_{DA}=\frac{1}{4d^{2}}{∥C_{s}-C_{t}∥}_{2}^{2}$$

(6) $$C_{s}=\frac{1}{N_{s}-1}\left(X_{s}^{T}X_{s}-\frac{1}{X_{s}}{\left(1^{T}X_{s}\right)}^{T}\left(1^{T}X_{s}\right)\right)$$

(7) $$C_{t}=\frac{1}{N_{t}-1}\left(X_{t}^{T}X_{t}-\frac{1}{X_{t}}{\left(1^{T}X_{t}\right)}^{T}\left(1^{T}X_{t}\right)\right)$$

(8) $$L=L_{classification}+\alpha L_{DA},\mathrm{where}\;0\leq \alpha \leq 1.$$

**Algorithm 1** Cloud-Based Retraining Algorithm
 1:Given a classification task with *C* categories.**Input:** 
Source features $f_{s_{i}}$ and some uploaded features $f_{t_{i}}$. 2:Perform data augmentation on $f_{t_{i}}$ and obtain $f_{new\_t_{i}}$. Use a portion of $f_{s_{i}}$ and $f_{new\_t_{i}}$ as the training set $X_{retrain}$ to train a teacher model. 3:**for** each epoch **do** 3: Obtain samples $x_{t}$ with a confidence level higher than $\gamma_{server}$. 3: Calculate the coral loss $L_{DA}$. 3: Calculate the total loss in Equation (8). 4:
**end for**
 5:**Output:** A new Teacher model $M_{s}$.


The teacher model is transferred to a new student model $M_{t}$ after retraining. The identical model compression method mentioned in Equation (4) is applied here. Beyer et al. [41] showed that knowledge distillation is more effective when the inputs to the teacher and student models are consistent, so $X_{retrain}$ is still used as the input to the student for distillation. The complete compression process is in Algorithm 2.
**Algorithm 2** Compression Algorithm**Input:** Use $X_{retrain}$ as the training set and a teacher model $M_{s}$. 1:**for** each epoch **do** 1: Calculate the soft loss $L_{soft}$. 1: Calculate the hard loss $L_{hard}$. 1: Calculate the total loss in Equation (4). 2:**end for** 3:**Output:** A new student model $M_{t}$.

#### 3.3.3. Data Distribution

The cloud requires computation of source domain features prior to being sent to edge nodes in order to provide a basis for them to determine whether to submit the data. However, storing all the source domain features is far beyond the memory capacity of an edge node in LPWAN. The cloud only calculates the normalized average vector ${\bar{d}}_{s_{i}}$ of each category in the source domain to conserve memory overhead. As for how the nodes use ${\bar{d}}_{s_{i}}$, this will be continued in Section 3.4.3.

### 3.4. The Perception Layer

**Figure 5:**
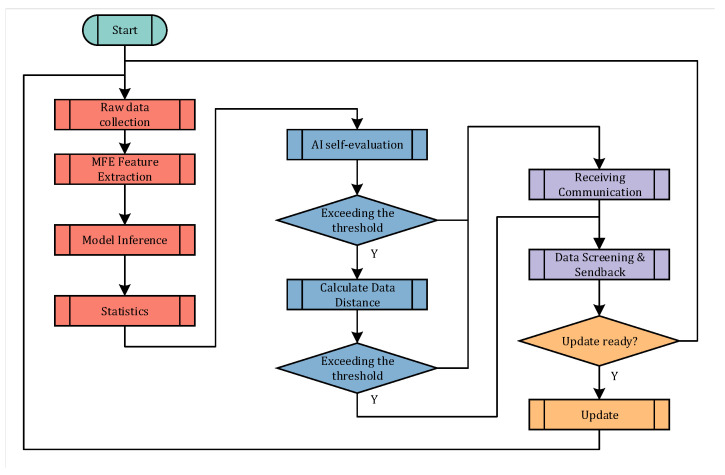
Node State Diagram. The leftmost boxes show regular tasks of the node, i.e., constantly collecting data for inference. The middle section displays the intelligent decision-making function specific to LPAI. Furthermore, the right section displays the data interaction process with the upper layer network.

Unlike traditional AIoT, the perception layer in LPAI handles more additional tasks. Therefore, edge nodes are supposed to schedule the tasks rationally within the limited resources. Figure 5 shows the task flow diagram of the edge node. The tasks are divided into three main parts:

- The edge node collects real-world data and performs inference.
- During each inference cycle, the edge node determines whether the current features after feature extraction needs to be sent back to the server.
- The edge node receives a new model and loads it into memory.

#### 3.4.1. Edge Intelligence

In LPAI, a neural network model is trained in the cloud using the Keras framework and then converted into hexadecimal data suitable for embedded devices with the help of Tensorflow Lite. This tool is used for deploying deep learning models on mobile and embedded devices, consisting of a converter and an interpreter. It also accomplishes network optimizations, such as quantization during the conversion process. The interpreter is responsible for transforming a .tflite file into a format that can be deployed on mobile devices and embedded microcontrollers. The hexadecimal model is then loaded into the device with the help of an open-source inference framework provided by Edge impulse to implement model inference.

#### 3.4.2. Feature Extraction

To significantly reduce the amount of data returned, important information needs to be extracted from the raw data. The traditional spectrogram method divides the window into multiple overlapping frames and then computes the FFT for each frame [42]. The size and number of frames can be adjusted with the parameters Frame length and Frame stride. For example, with a window of 1 s, frame length of 0.02 s and stride of 0.01 s, it will create 99 time frames. An FFT is then calculated for each frame. Finally, the noise floor value is applied to the power spectrum. In edge impulse, to adapt to non-voice data, two additional steps are added. After computing the spectrogram, a triangular filter is applied on the Mel scale to extract frequency bands. The main idea is to extract more features in low and less in high frequencies. The final step is to perform local mean normalization of the signal, applying the noise floor value to the power spectrum.

#### 3.4.3. Data Screening Algorithm

**Figure 6:**
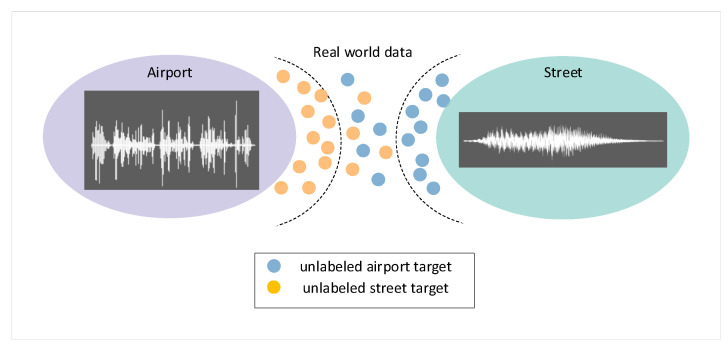
Taking binary classification as an example, the labeled source domain data can be classified by the class boundary, but in the target domain, the data closer to the class boundary has a higher probability of being correctly classified, while the data farther away from the class boundary is difficult to distinguish. Assigning pseudo-labels to all target data generates numerous errors.

The edge nodes of LPAI can screen representative features autonomously. As mentioned above, the average vector ${\bar{d}}_{s_{i}}$ is pre-stored in the node as a baseline for the source domain data. Y. Kim et al. [43] mentioned that the pseudo-labels are more accurate for data closer to the source domain, while the opposite is true for data farther away from the source domain, as shown in Figure 6. The algorithm of Yang et al. and Y. Kim et al. [31,43] cannot be directly applied to LPWAN because of the limitations of embedded devices. Hence, a more ingenious implementation is designed.

In each round of inference, the edge node collects raw acoustic data through sensors, acquires $f_{t_{j}}$ after feature extraction, and then infers the classification result by forward propagation. The node decides whether to upload the feature if the confidence level is higher than $\gamma_{nodes}$. $\gamma_{nodes}$ can be interpreted as the probability that the node has $\gamma_{nodes}$ to trust its judgment. It should be set lower than $\gamma_{server}$ because the features that confused the nodes should be selected and retrained.

Next, the distance $d_{f_{j}}$ between $f_{t_{j}}$ and each ${\bar{d}}_{s_{i}}$ is calculated. However, an edge node cannot record each $f_{t_{j}}$ and sort them based on the distance as Y. Kim et al. did. Even storing the reduced-dimensional features is still impossible for low-power devices. Therefore, a more straightforward approach is taken to find the target features that confuse the nodes. It is to design a temporary variable $d_{temp}$, which is the average of $d_{f_{j}}$. It represents the average distance of all the target domain data from the source domain. Whenever a edge node obtains a $f_{t_{j}}$, it caculates the distance $d_{f_{j}}$ and update $d_{temp}$. To ensure the correctness of the pseudo-labeling while electing features confused the nodes, the final features to be uploaded are those near $d_{temp}$, which is to satisfy Equation (9). Because the number of newly data is as few as possible, η is as close to 0 as possible. The judgment process is shown in Algorithm 3. Its computational complexity is O(n), where *n* is the total number of data points in the target domain.

(9) $$\left(1-\eta \right)\times d_{temp}<d_{f_{t_{i}}}<\left(1+\eta \right)\times d_{temp}$$

**Algorithm 3** Data Screening Algorithm On the Node
 1:Given ${\bar{d}}_{s_{i}}$ from the server, *i* = 1 …*C*, where *C* is the number of categories.**Input:** A new target data: 2:Obtain $f_{t_{j}}$ after feature extraction. 3:Perform inference. 4:**if** Its confidence level is greater than $\gamma_{nodes}$ **then** 5:   **for** each $f_{t_{j}}$ **do** 5:  Calculate the distance between each $f_{t_{j}}$ and the ${\bar{d}}_{s_{i}}$ of the corresponding pseudo-label category. 5:  Update the average distance $d_{temp}$ once. 6:  **if** Equation (9) is satisfied **then** 7:   **Output:** upload $f_{t_{j}}$. 8:  **end if** 9: **end for** 10:
**end if**



## 4. Evaluations and Results

**Figure 7:**
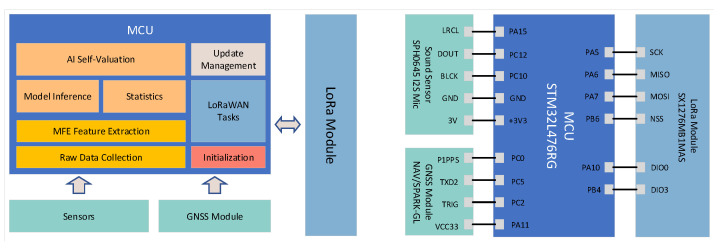
A simple memory management diagram for edge nodes.

In this section, we conduct extensive experiments to demonstrate the ability of LPAI to enable edge nodes to enhance their capabilities in an environment based on few-shot data. We validate it with three acoustic scene classification datasets using LoRa, a typical network in LPWAN. Experiments are based on a four-classification scenario. We have deployed LoRa nodes at a school intersection to monitor the operation of shuttles. Its hardware diagram is shown in Figure 7.

The evaluation is divided into three parts: the first part evaluates the effectiveness of the cloud-based retraining phase. It is assumed that all target domain features have been uploaded, so the model is trained with the source domain data and a portion of the target domain data and also tested on the remaining target domain data. Moreover, the second part evaluates the effectiveness of Algorithm 3 for screening the dataset. The results of uploading all target data and the random screening method are used as benchmarks. Suppose the model is retrained after collecting 85 points of data to mimic the operation pattern of nodes in the environment. In the last section, we combine experimental and theoretical analysis to explore the impact on LoRa network performance.

### 4.1. Datasets

- TAU Urban Acoustic Scenes 2020 Mobile, development dataset [44]. It contains recordings from 12 European cities in 10 different acoustic scenes using 4 different devices. Because only four-classification questions are discussed, four scenarios commonly used in LPWAN are chosen: metro, metro station, street pedestrian and traffic. However, changes in the environment can lead to differences between domains, and the evaluation is divided into five domains according to the collection location, namely A1 (Barcelona, Helsinki), B1 (Lisbon, London), C1 (Milan, Lyon), D1 (Paris, Prague), E1 (Stock, Vienna). This dataset is divided into several source-domain and target-domain datasets according to different locations. The baseline system for the 2020 DCASE Challenge provides an accuracy of 54.1%.
- Urban sound 8K [45]. The dataset contains 8732 labeled sound excerpts from 10 categories of urban sounds. Take four of them: marked as A2. Refer to paper [45] for baseline results.
- Dataset AOB [46]. This dataset uses a convolutional neural network to collect and manually edit an audio dataset for urban sound event classification for a master’s thesis. Take four of them: marked as A3.

After experiments, we discovered that an edge node could recognize a dataset up to 2 s in length in our scheme. Considering power consumption, recognition performance and other factors, the audio length was determined to be 1 s. Therefore, the above three datasets were downsampled to 16 kHz and cut into 1-second fragments before training. After feature extraction, the original dataset with a length of 1 s and a size of 64K bytes were compressed into feature data with 4K bytes. This means that the quantity of data transmitted over the network is compressed by a factor of 16.

### 4.2. Setup and Implementation Details

#### 4.2.1. Evaluating Cloud-Based Retraining Algorithms

**Figure 8:**
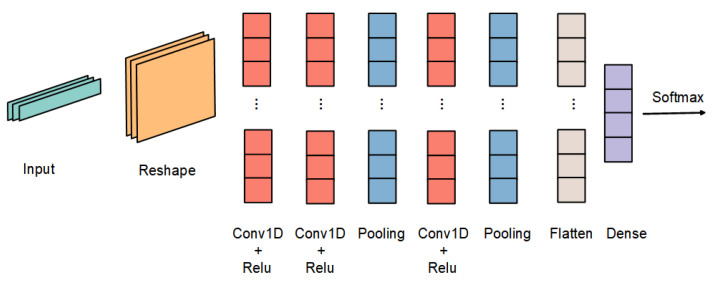
Teacher model structure. This structure comprises multiple 2D convolutional layers, pooling layers, and dropout layers. The convolutional layer represents the core part of recognition and is used to extract different features from the data. The pooling layer is used to reduce information redundancy and model computation, reduce the difficulty of network optimization, and prevent network overfitting.

In this section, we evaluate the effectiveness of the retraining phase. We first train an initial teacher model with the source domain data to evaluate its performance on the target domain, providing a baseline result. The structure of the teacher model is shown in Figure 8. We use 80% of the source domain as the training set and test the remaining 20%. The reason for designing this experiment is to demonstrate that the domain-shift problem exists and to provide a baseline for subsequent experiments to prove the validity of our approach.

Next, we evaluate the effectiveness of Algorithm 1. We assume that all unlabeled target domain data are uploaded to the cloud. Unlike the traditional approach, we use 80% of the unlabeled data as the training set and 20% as the test set. The parameters are set as follows, $\gamma_{nodes}$ is 0.5, and $\gamma_{server}$ is 0.7. Adam is employed as the optimizer. Its learning rate is 0.0001, $\beta_{1}$ is 0.9, $\beta_{2}$ is 0.99, and the iteration epoch is 20.

Subsequently, we evaluate the effectiveness of the cloud-based retraining phase by assuming all target domain feature data are uploaded to the cloud. The reason for this design is firstly to evaluate that our method can reduce the bias between different domains, and secondly to provide a control for subsequent evaluation of the data screening algorithm. Therefore, the inputs to this part of the algorithm are all labeled source domain features and all unlabeled target domain features. Unlike the traditional approach, we use 80% of the unlabeled data as the training set and 20% as the test set. The parameters are set as follows: $\gamma_{nodes}$ is 0.5, and $\gamma_{server}$ is 0.7. Adam is used as the optimizer with a learning rate of 0.0001, $\beta_{1}$ is 0.9, $\beta_{2}$ is 0.99, and the iteration period is 20. The output of the algorithm is a teacher model with optimal performance.

**Figure 9:**
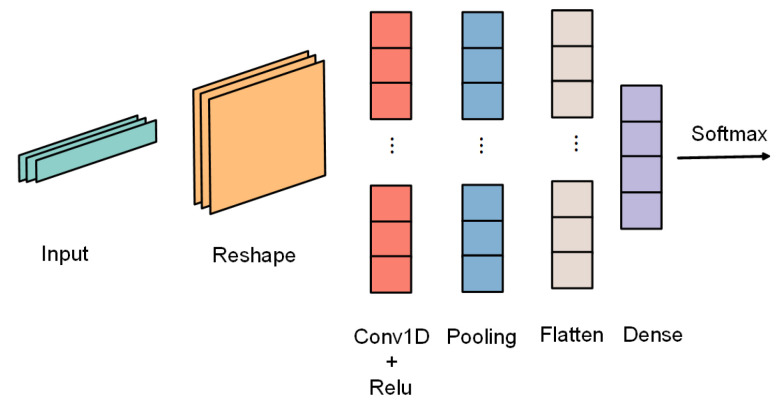
Student model structure. This structure consists of two-dimensional convolution, pooling and dropout layers, with a smaller number of parameters than the teacher model and suitable for low-power embedded devices.

Finally, the performance after compression was evaluated. In this part, the input of the student model is the same as in the previous step, i.e., the input of the teacher model, and after iterative training, an optimal student model is obtained. In addition, the student model is finally updated to the edge node. The structure of the student model is shown in Figure 9.

The results of the first part of the experiment are presented in Table 2. All results are measured according to two criteria: accuracy and Kappa coefficient. Accuracy is the most common metric for classification problems and is used to measure the model’s accuracy on the test set. The Kappa coefficient is commonly used for multi-classification issues and is employed to measure consistency. Consistency refers to whether the model predicts the same results as the actual classification results. It usually lies between 0 and 1, and a larger Kappa coefficient indicates greater consistency. The experiments were conducted on three data sets, and group A1–B1 means that A1 is the labeled source domain and B1 is the unlabeled target domain data. The first two columns record the model performance trained with the source domain data on the source test set and the target domain. The performance of the source model on different target domains is recorded to facilitate comparison with the results of subsequent experiments. The last three columns show the performance after our cloud-based retraining algorithm. The performance of the model in the source domain, the target domain, and the student model in the target domain after retraining is recorded, respectively. The last column records the performance of the model deployed on the edge nodes after compression. Taking group A1–B1 as an example, we can see from S (before DA) and T (before DA) that, as expected, the performance of the model trained on the source domain data degrades on the target domain data, which indicates that the domain-shift problem arises from changes in the environment. After retraining, the data of S (after DA) are generally better than S (before DA), which means that the performance of the model does not degrade, or indeed even slightly increases on the source domain data. Furthermore, the performance of the target domain data is also greatly improved. That is, our approach makes the recognition performance of edge nodes better. Finally, the recognition accuracy remains the same before and after compression from the last column. Most importantly, a model of this size can be easily deployed on embedded devices. Table 3 shows the time and space complexity. The time complexity of the model is measured by floating point operations (FLOPs), and the space complexity is measured by the number of model parameters. The compression ratio is approximately 0.4.

#### 4.2.2. Evaluating Data Screening Algorithms

**Figure 10:**
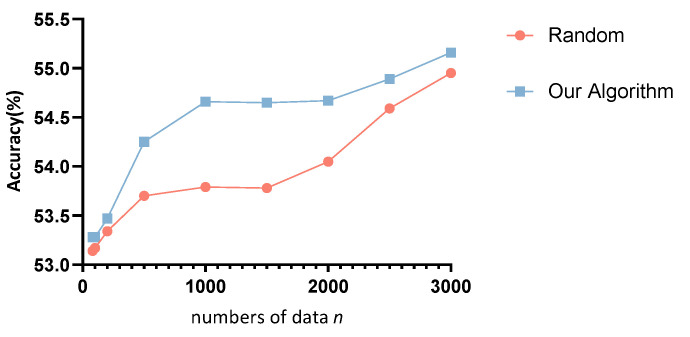
Randomly filter *n* pieces of data and use them to retrain the corresponding accuracy.

The effectiveness of the algorithm for screening the datasets is then evaluated. To highlight that our algorithm can select data that are more useful for model updates, we designed a random screening method and Algorithm 3 for comparison, where *n* data were randomly screened on the target domain data. We screened three times and took the average value with the same amount of data to ensure fairness and randomness. The results are shown in Figure 10 for A1–D1 group of the TAU dataset. We also evaluated the effect of the data augmentation method. In Figure 10, the blue line represents the performance of our algorithm. By default, 85 pieces of data are filtered by adjusting η in Equation (9), and then the target domain is expanded to the corresponding *n* using the data multiplication method.

In addition, to emphasize the ability of our algorithm to render the nodes more applicable to the environment within the constraints of LPWAN, three control groups were set up to evaluate the effectiveness of Algorithm 3. The first one is the accuracy of the source domain model in all target domain data before retraining, the second one is the accuracy of the new model in the only 85 pieces of uploaded target features after implementing Algorithm 3, and the third one is the accuracy after uploading all target domain features.

**Figure 11:**
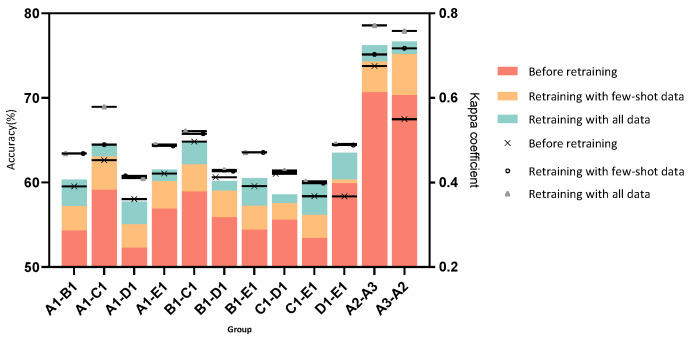
Data screening algorithm evaluation results. The bar chart indicates the accuracy before and after training, and the scatter plot indicates the Kappa before and after training. Each evaluation criterion is compared with three control groups, the first one is the accuracy of the model in the target domain without retraining, the second one is the result of uploading only 85 data points from the Algorithm 3, and the last one is the result of uploading all target domain features.

Figure 10 and Figure 11 show the performance of the data screening algorithm. In Figure 10, the accuracy steadily increases as *n* increases, implying that our algorithm performs better compared to random screening and can sort out higher quality data with the same amount of data. The effectiveness of the data augmentation method can still be seen from the figure, where the accuracy increases gradually as *n* increases after 85 s data are uploaded. In Figure 11, the effectiveness of our method can be clearly seen from the three control groups. On the one hand, uploading only 85 s of data collected from one node can significantly improve the recognition performance compared to not performing updates (before retraining in Figure 11). On the other hand, similar results are achieved in both evaluation criteria of accuracy and Kappa coefficient compared to uploading tens of thousands of data points. In particular, in some groups, such as A3–A2, the accuracy is improved by 5% and the Kappa coefficient by 0.17 compared to uploading all data. While there are still gaps with regard to uploading all the target features, these gaps are insignificant compared to the consumption of resources such as power. This is also in line with the usage scenario of low-power IoT.

#### 4.2.3. Evaluating Network Performance

Finally, we evaluate the network performance of the LPAI framework and its impact on the network in the standard LoRaWAN protocol through experiments and theoretical calculations, including the response time and the time required for model updates.

LPAI’s network response time. Our nodes support two application modes of edge intelligence.

**Figure 12:**
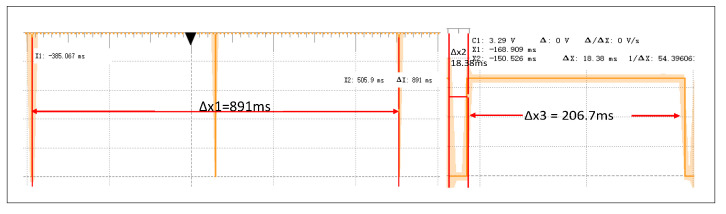
Response time for each task of the edge node. In the left figure, $\Delta x_{1}$ represents the time taken by the sensor to collect 0.5 s of data, in the right figure, $\Delta x_{2}$ represents the model inference time, and $\Delta x_{3}$ represents the time taken by the data screening algorithm.

The first is the one-shot recognition mode. Namely, edge nodes perform inference and data screening only once. The process includes 1. Data acquisition: From collecting raw data to encoding using digital audio sensors. 2. Model inference: After extracting the raw data by MFE features, the classification result is then propagated by a neural network. 3. Data screening. The data screening algorithm determines whether these data need to be sent to the cloud. We use an Analog Discovery 2 oscilloscope to calculate the time required for each stage by flipping the I/O pin level, and the result is shown in Figure 12. The result is divided into three parts, where the first part is the process of obtaining raw data collected by the sensor. For continuous acquisition, the microcontroller acquires voice data in DMA dual mode, each buffer holds 0.25 s of audio data, and the double buffer pointers are exchanged each time one of the buffers fills up. The 0.25 s of data collected is appended to the MFE’s operation window, and subsequent inference operations are performed when the window is filled with 1 s of data. The second part is to perform model inference after feature extraction from the original data. The third part judges whether to upload the compression feature through the data screening algorithm. The experimental results show that for one second of acoustic data, the total execution time is about 2 s.

**Figure 13:**
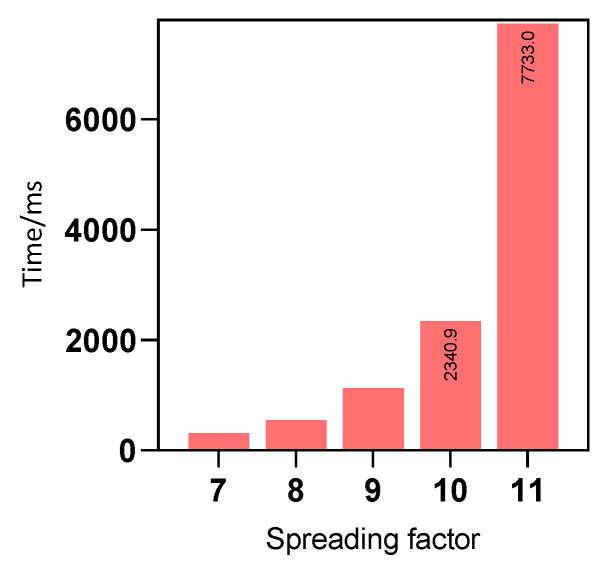
The LoRa node sends a 180-byte response time on the CN470 band.

The second is the periodic recognition mode: the edge nodes are continuously recognized at a fixed sampling interval over a long period. That is, the edge nodes count events continually within a day. Take the scenario of identifying a vehicle at an intersection as an example. Each event is expressed as a 1-bit Boolean value. Assuming that in the most extreme case: uploading statistics for every minute within one day requires 1440 bits of data. Therefore, the total payload uploaded per day is about 180 bytes. Using class C of the LoRaWAN protocol for theoretical calculation, the time $T_{tx}$ required to upload 180 bytes in the CN470 frequency band is shown in Figure 13 and calculated by Equation (10) [47]. $T_{tx}$ is composed of two parts: the preamble and the payload, calculated as Equations (11) and (12), where SF denotes the spreading factor, BW is the bandwidth, $N_{pre}$ is the preamble length, $N_{PHY}$ is the payload length, CR denotes the coding rate which can take values from 1 to 4, and PL is the payload rate that indicates the physical payload length in bytes. It can be seen from the figure that as the spreading factor increases, the data rate decreases, and the transmission time of each data packet increases. When the spreading factor is 11, the response time is 7733 ms, which requires approximately 13 min with a 1% duty cycle.

(10) $$T_{tx}=T_{preamble}+N_{PHY}\times \frac{2SF}{BW}$$

(11) $$T_{preamble}=\frac{2SF}{BW}\times \left(N_{pre}+4.25\right)$$

(12) $$N_{PHY}=8+\max\left[ceil\left[\frac{28+8PL+16\times CRC-4SF}{4\times (SF-2DE)}\right]\times \left(CR+4\right),0\right]$$

Time to back propagate compressed features and model updates. LPAI only needs to upload 85 compressed features to ensure 5% accuracy improvement, and each compressed feature occupies 4k bytes. Since LoRaWAN has a constraint on the payload length of a single packet, in the worst case, where the SF is 11, the maximum payload length is 223 bytes, so a compressed feature requires 18 packets to be sent out. According to Equation (10), the time to backhaul a single 4KB dataset is about 10 min at a maximum duty cycle of 1%. Furthermore, for the model update part, consider the worst case, which is 30k bytes at a time, where the time required at an SF of 7 is 83 min. In these calculations, we assumed continuous packet transmission. In a real-world scenario, LoRa nodes only upload a few pieces of data per day. Therefore, complete data are only sent down after weeks or even months, and that amount matches the LPWAN scenario.

## 5. Conclusions

In this paper, we propose LPAI, the first generic LPWAN-based AIoT framework for acoustic scene classification. We demonstrate experimentally in LoRa networks that the recognition accuracy of LoRa nodes can be improved by 5% by uploading only 85 compressed data points. In addition, we analytically prove the feasibility of LPAI, which bears important practical implications. LPAI advances LPWAN to a new frontier. In future research, we will further improve LPAI. On one hand, we will evaluate other standard networks for LPWAN, such as sigfox, with longer transmission distances and more stringent conditions. On the other hand, LPAI should also be applied to the imaging-based fields to promote LPAI as a more general AIoT system.

## Figures and Tables

**Table 1:** The latest research on AIoT frameworks and the differences in LPAI.

Reference	Proposed	Based on LPWAN	Rely on the Backbone Network	Consider Model Update
[16]	An open-source deep learning training and inference framework	×	✓	×
[17]	A deep learning model for human pose estimation and tracking	×	✓	×
[18]	A collaborative computing platform between edge devices and the cloud	×	✓	✓
[9]	A system to monitor bat species based on echolocation audio.	✓	×	×
[19]	An AIoT precision feeding management system to measure water surface fluctuations in areas of fish pellet application	✓	×	×
[20]	An intelligent-assistance system for visually impaired people to achieve the goals of aerial obstacle avoidance and fall detection.	✓	×	×
[33]	A wearable assistive system to help visually impaired consumers safely use marked crosswalks	✓	✓	×
[34]	A flood observation system based on LoRa to integrate with the Internet	✓	✓	×
LPAI	A generic framework for implementing AIoT technology based on LPWAN applicable to the acoustic scene classification (ASC) scenarios	✓	×	✓

**Table 2:** The training results in the source and target domains, where S represents the test results of the teacher model in the source domain, T represents the results of the teacher model in all target domain data, and stu accuracy denotes the results of the student model, acc denotes the accuracy, and K is the Kappa coefficient.

	S (before DA)		T (before DA)		S (after DA)		T (after DA)		stu	
	acc	K	acc	K	acc	K	acc	K	acc	K
A1–B1	66.48	0.5506	54.30	0.3906	69.95	0.5671	60.34	0.4688	60.08	0.4685
A1–C1	66.48	0.5506	59.12	0.4529	70.49	0.6183	64.46	0.5791	64.38	0.5790
A1–D1	66.48	0.5506	52.27	0.3606	66.79	0.5847	57.56	0.4102	57.76	0.4123
A1–E1	66.48	0.5506	56.88	0.4213	70.04	0.6092	62.17	0.4900	61.05	0.4853
B1–C1	66.98	0.5905	58.92	0.4969	69.74	0.6169	64.78	0.5217	62.34	0.5207
B1–D1	66.98	0.5905	55.91	0.4121	71.75	0.6136	60.17	0.4297	60.52	0.4304
B1–E1	66.98	0.5905	54.42	0.3915	71.59	0.6050	60.52	0.4712	60.35	0.4710
C1–D1	73.27	0.6409	55.59	0.4204	73.66	0.6833	58.60	0.4284	58.91	0.4302
C1–E1	73.27	0.6409	53.43	0.3675	73.79	0.6281	59.96	0.4022	59.97	0.4020
D1–E1	65.54	0.5444	59.91	0.3672	67.71	0.5641	63.53	0.4914	63.51	0.4915
A2–A3	76.32	0.8625	70.68	0.6754	79.13	0.8649	76.23	0.7714	76.41	0.7728
A3–A2	75.42	0.8160	70.32	0.5499	84.77	0.8158	76.68	0.7584	76.92	0.7613

**Table 3:** Algorithmic complexity of neural networks. Floating point operations (FLOPs) represent the time complexity and the number of model parameters measures the space complexity.

Model	FLOPs	Parameter Quantity
Teacher	3304	3340
Student	1762	1388

## Data Availability

https://www.freepik.com/free-vector/sound-waves-collection_1014327.htm (accessed on 28 November 2022).

## Abbreviations

The following abbreviations are used in this manuscript:

AI	Artificial Intelligence
IoT	Internet of Things
AIoT	Artificial Intelligence of Things
LPWAN	Low–Power Wide Area Network
DA	Domain Adaptation
DCNN	Deep Convolutional Neural Network
ASC	Acoustic Scene Classification
SF	Spreading Factor
